# Psychological Distress after a Diagnosis of Malignant Mesothelioma in a Group of Patients and Caregivers at the National Priority Contaminated Site of Casale Monferrato

**DOI:** 10.3390/ijerph17124353

**Published:** 2020-06-17

**Authors:** Michela Bonafede, Antonella Granieri, Alessandra Binazzi, Carolina Mensi, Federica Grosso, Gianluca Santoro, Isabella Giulia Franzoi, Alessandro Marinaccio, Fanny Guglielmucci

**Affiliations:** 1 Occupational and Environmental Medicine, Epidemiology and Hygiene Department, Italian Workers’ Compensation Authority (INAIL), 00143 Rome, Italy; a.binazzi@inail.it (A.B.); a.marinaccio@inail.it (A.M.); 2 Department of Psychology, University of Turin, 10123 Turin, Italy; antonella.granieri@unito.it (A.G.); isabellagiulia.franzoi@unito.it (I.G.F.); fanny.guglielmucci@unito.it (F.G.); 3 Epidemiology Unit, Fondazione IRCCS Ca’ Granda, Ospedale Maggiore Policlinico Milano, 20122 Milan, Italy; carolina.mensi@unimi.it; 4 Mesothelioma Unit, Azienda Ospedaliera SS Antonio e Biagio e Cesare Arrigo, 15121 Alessandria, Italy; federica.grosso@ospedale.al.it; 5 Faculty of Human and Social Sciences, UKE—Kore University of Enna, 94100 Enna, Italy; glcsnt@gmail.com

**Keywords:** malignant mesothelioma, emotional distress, contaminated site, depression, trauma, post-traumatic stress disorder (PTSD)

## Abstract

Background: Patients of malignant mesothelioma (MM) and their caregivers face significant physical and psychological challenges. The purpose of the present study is to examine the emotional impact after the diagnosis of MM in a group of patients and familial caregivers in a National Priority Contaminated Site (NPCS). Methods: A sample of 108 patients and 94 caregivers received a sociodemographic/clinical questionnaire, the Beck Depression Inventory II, the Davidson Trauma Scale, the Coping Orientation to the Problems Experienced—New Italian Version, and the Defense style questionnaire. The risk of depressive and post-traumatic stress disorder (PTSD) symptoms in relation to the strategies of coping and defense mechanisms was estimated in patients and caregivers separately by logistic regression models. Results: For patients, a high risk of depression was associated with high usage of Defense Style Questionnaire (DSQ) Isolation (OR: 53.33; 95% CI: 3.22–882.30; *p* = 0.01) and DSQ Somatization (OR: 16.97; 95% CI: 1.04–275.90; *p* = 0.05). Other significant risks emerged for some coping strategies and some defenses regarding both depression and trauma in patients and caregivers. Conclusions: This research suggests that for both patients and caregivers unconscious adaptive processes have a central role in dealing with overwhelming feelings related to the disease.

## 1. Introduction

The impact of asbestos is currently a serious concern for public health. Recently, Furuya et al. [1] summarized the latest evidence on the magnitude of asbestos-related diseases, showing a global estimate of 255,000 deaths/years (243,223–260,029) due to asbestos. Moreover, work-related exposures are responsible for 233,000 deaths (222,322–242,802). Along with the progressive growth of these occurrences, Malignant Mesothelioma (MM) death numbers are also increasing constantly, reaching a global total of 30,208 cases, 91.4% of those due to occupational exposure [1], mostly in men.

Due to the large use of asbestos before the national ban in 1992 [2], Italy is one of the countries most involved in the monitoring and control of asbestos-related diseases. Currently, Italy is suffering a severe epidemic of asbestos-related diseases. For this reason, Italy has implemented an epidemiological surveillance system of mesothelioma incidence through a national registry (ReNaM: Registro Nazionale dei Mesoteliomi) with the aim to provide evidence regarding pleural mesothelioma occurrence and the modalities of asbestos exposure involved. More than 27,000 malignant mesothelioma cases have been collected from 1993 to 2015, with exposures defined for around 70%. Environmental and domestic exposures have been reported in 4.4% and 4.9% of cases, respectively [3].

Mesothelioma incidence has been investigated at several National Priority Contaminated Sites (NPCSs) in Italy in order to obtain estimates on the impact of asbestos on residents. Statistically significant excesses of mesothelioma incidence were recorded at 20 of these sites, including Casale Monferrato, where a large fiber cement factory (i.e., Eternit) is headquartered. The factory has had an impact on the health not only of workers employed at the plant (mostly men), but also that of their relatives (mostly wives) and other residents, with no other sources of asbestos exposure besides environmental pollution [4].

The standardized incidence ratios (SIR) of MM in Casale Monferrato are 910.7 (95% CI: 816.5–1012.8) in men and 1338.1 (95% CI: 1176.7–1515.4) in women [5].

The consequences of MM on mental health are particularly relevant in National Priority Contaminated Sites (NPCSs). Our previous studies in the NPCS of Casale Monferrato have remarked negative mental health outcomes in the whole residing population [6,7].

MM patients and their familial caregivers face significant physical and psychological challenges, such as a limited life expectancy, and poor and fatal prognosis [8]. The lack of effective treatments, together with the difficulty of communicating with physicians, underlies the increased incidence of intense emotional disturbance resulting from the diagnosis of MM [9]. Indeed, the literature has remarked that the diagnosis of MM is a shocking traumatic experience associated with depressive symptoms and feelings of hopelessness, helplessness and despair [10,11,12,13,14]. A depressed state is a common psychopathologic feature of MM [15,16,17] that worsens with disease progression [18]. Asbestos exposure has also been linked to post-traumatic symptomatology [18,19], presumably related to the awareness and fear of imminent death [6,20,21]. 

Humans cope with adversity and negative emotions in adaptive and non-adaptive ways. Coping strategies and defense mechanisms are psychological processes that allow an individual to manage painful emotions arising from stressful and traumatic experiences. However, they differ in several fundamental aspects. Coping is a conscious and intentional process for managing specific internal and external demands that are believed to exceed the resources of a person. In contrast, defense mechanisms are mainly unconscious and unintentional, and aim to protect the ego from the intensity of disruptive internal/external conflicts and dangers [22]. Moreover, coping is related to an individual level of adaptation and is externally determined by a situation that leads to change, whereas defense mechanisms are thought to be related to personality traits and are thus stable over time, as well as being internally determined and proportional to the degree of reality or distorted perception [23].

Previous studies have shown that in order to face the traumatic emotions linked to MM diagnosis and its symptoms, MM patients commonly activate avoidant strategies which involve cognitive and behavioral efforts to minimize the real health danger of asbestos exposure [9,15,21,24]. Additionally, psychoanalytic in-depth investigations have highlighted the tendency of both MM patients and their caregivers to move away from conscious thoughts and emotions related to death through denial [13].

The purpose of the present study was to examine the emotional impact (depression and trauma) of a diagnosis of MM in a group of patients and familial caregivers in the NPCS of Casale Monferrato with a special focus on the coping strategies and defense mechanisms that are used by these two groups.

## 2. Materials and Methods

### 2.1. Procedures and Study Population

The present study was part of the Assessing Psychological Variables in Mesothelioma (AVPM) Project, conducted at the Mesothelioma Unit of Casale Monferrato-Alessandria, Italy. The protocol was approved by the local Institutional Review Board of the University of Turin and the Azienda Ospedaliera SS Antonio e Biagio e Cesare Arrigo Ethical Committee (AVPM; 14/11/2014). The study was carried out in accordance with the Declaration of Helsinki. All subjects provided written informed consent and their anonymity was ensured.

The present study followed a cross-sectional design, having continuous recruitment between September 2014 and December 2018. Cases were selected from the medical database of the Mesothelioma Unit based on the following inclusion criteria: diagnosed with MM or being a primary familial caregiver of an MM patient, the ability to speak Italian, living in in the NPCS of Casale Monferrato and providing written informed consent. Exclusion criteria were as follows: diagnosis of a psychiatric illness or central nervous system disease, and having a caregiver with a disease related to asbestos exposure.

Potential participants were recruited through an invitation from medical staff (i.e., oncologists, nurses and psychologists) at the site, who introduced the study and gave the participants an information sheet. The response rate for both patients and caregivers was 80%. In the recruitment phase, the two groups were unrelated and independent. Patients were recruited within 6 months of diagnosis. Researchers were not involved in patient care, but trained medical staff in recruitment and test administration, which was carried out by hospital psychologists in an individual setting. Data were anonymously and independently analyzed by researchers.

### 2.2. Instruments

#### 2.2.1. Sociodemographic and Clinical Data Collection

An ad hoc questionnaire was used to collect sociodemographic and clinical variables including: gender, age, educational level, job, marital status, psychotherapy history, psychological examination, drugs use, type of MM, and localization. Asbestos exposure (environmental/occupational) determined through a ReNaM questionnaire (for patients only).

#### 2.2.2. Depression

The Beck Depression Inventory, Second Edition (BDI-II) [25] is a 21-item self-report questionnaire assessing the presence and severity of depressive symptoms according to the Diagnostic and Statistical Manual of Mental Disorders, Fourth Edition (DSM-IV) criteria. Scores equal to or under 13 inform the absence of or minimal depression; scores between 14–19 and 20–28 indicate mild and moderate depression, respectively; while scores equal to or above 29 indicate severe depression. 

The items refer to sadness, pessimism, a sense of failure, self-dissatisfaction, guilt, self-punishment, self-deprecation, self-accusation, suicidal ideas, crying, irritability, social withdrawal, indecisiveness, body image distortion, difficulties at work, sleep disturbance, fatigue, loss of appetite, weight loss, preoccupation, and decreased libido. All but two items have four possible responses of increasing intensity. For example, under sadness the responses range from “I do not feel sad” to “I am so sad or unhappy I can’t stand it”. The range of possible scores is 0–63. The degree of a symptom was measured with a four-item Likert-type scale reflecting the presence or frequency of the symptom.

The BDI-II has been translated into several languages and adapted to different cultures, and its utility has been demonstrated in many studies [26]. The Italian version of the scale demonstrated good internal consistency and concurrent validity [27].

#### 2.2.3. Post-Traumatic Symptoms

The Davidson Trauma Scale (DTS) [28] is a 17-item self-report questionnaire assessing the frequency and severity of each DSM-IV symptom of post-traumatic stress disorder (PTSD). In the present study we modified the original version (i.e., record “the trauma that is most disturbing to you”), asking participants to answer items already referring to the trauma of MM diagnosis.

Items were rated on five-point frequency (0 = “not at all” to 4 = “every day”) and severity scales (0 = “not at all distressing” to 4 = “extremely distressing”). Respondents were asked to identify the trauma that is most disturbing to them and to rate, in the past week, how much trouble they have had with each symptom (sample item “Have you had painful images, memories or thoughts of the event?”). The DTS yields a frequency score (ranging from 0 to 68), severity score (ranging from 0 to 68), and total score (ranging from 0 to 136).

Items can be categorized into three clusters corresponding to the DSM-IV diagnostic criteria for PTSD diagnosis: Criteria B “re-experimentation” (RE; items 1–5); Criteria C “avoidance and numbing” (AN; items 6–12); Criteria D “hyper-activation” (HA; items 13–17). Further research has shown that DTS could be used to differentiate patients with PTSD and partial PTSD from those with no PTSD in the general population, and to detect differences between treatments of differing efficacy [29]. DTS has been used in several previous studies to detect PTSD in Italian population [30,31].

#### 2.2.4. Coping Strategies

The Coping Orientation to Problems Experienced—New Italian Version is a 60-item self-report questionnaire assessing the frequency of strategies usually activated to cope with stressful events on a four-point scale (1–4) [32]. Response choices are the following: “I usually do not do this at all”, “I usually do this a little bit”, “I usually do this a medium amount”, and “I usually do this a lot”. Example statements from the inventory include “I concentrate my efforts on doing something about it” and “I take additional action to try to get rid of the problem”.

In the present study, we asked participants to answer the questions by considering the MM diagnosis as the stressful event. In contrast to the original version, the new Italian version has a five-factor structure (social support, avoidance strategies, positive attitudes, problem solving and turning to religion) [33]. The dimensions were large and essentially independent, with good internal consistency (alpha range 0.70–0.91; mean inter-item correlation range: 0.13–0.15) and stability (test-retest range 0.68–0.93).

#### 2.2.5. Defense Mechanisms

The Defense Style Questionnaire (DSQ-40) [34] is a 40-item self-report questionnaire widely used to assess defense mechanisms that is derived from the original version developed by Bond and colleagues [35]. Participants rate each item on a nine-point Likert scale ranging from 1 (strongly disagree) to 9 (strongly agree). This instrument investigates 20 defense mechanisms (sublimation, humor, anticipation, suppression, withdrawal, pseudo-altruism, idealization, reaction formation, projection, passive aggressive behavior, acting-out, isolation, devaluation, autistic fantasy, denial, displacement, dissociation, splitting, rationalization and somatization) [36]. Defenses are categorized into three ordered domains which represent defense styles: mature (eight items e.g., “I’m able to keep a problem out of my mind until I have time to deal with it”), neurotic (eight items e.g., “If I have an aggressive thought, I feel the need to do something to compensate for it”), and immature (24 items e.g., “If my boss bugged me, I might make a mistake in my work or work more slowly so as to get back at him”). The findings of the factor structure of the DSQ-40 are inconsistent, showing three-, four-, and five-factor solutions; for this reason, in the present study we did not use a categorization of defenses [37,38,39,40,41]. The DSQ-40 has been validated in many countries, including Italy [37].

### 2.3. Data Analysis

Descriptive statistics were used to characterize the sample in terms of sociodemographic and clinical variables. The chi-squared test (*χ*^2^) and analysis of variance (ANOVA) were used to evaluate differences between patients and caregivers, and gender. The homogeneity of variance was verified with the Levene test. The Brown–Forsythe and Welch tests were used when the homogeneity of variance assumption did not hold for the data. The mean ages were compared with the Student’s *t*-test for independent samples. 

The risk of depressive and PTSD symptoms in relation to coping strategies and defense mechanisms was separately estimated in patients and caregivers. Logistic regression models were used to explore significant factors associated with the two outcomes: depression (BDI) and PTSD symptoms (DTS). This was achieved by treating the effective response as the dependent variable and other factors as independent variables, which were introduced into the model as dichotomous ordinal variables and scored according to each respective cut-off, or to the mean for the five-factor “coping” structure. According to the original cut-offs proposed by Beck et al. [25], “13” was chosen as the end point for the BDI-II (absence or presence of depressive symptoms), while “40” was selected for the DTS (absence or presence of post-traumatic symptoms) [28,42]. Full and stepwise models were used to identify the combination of independent variables giving the best explanation of the outcome (using the overall predictive accuracy and R^2^ statistic). As measures of association, odds ratios (OR) and their 95% confidence intervals (CI) were reported. ORs greater than 1 indicated higher risks for depression and post-traumatic stress of being patient/caregiver or male/female or a strategy of coping and defense mechanisms activated, according to the model. The results were considered significant at a *p*-value less than 0.05. All analyses were performed using SPSS v25.0 for Windows (IBM, Armonk, NY, USA).

## 3. Results

The sample (Table 1) comprised 108 patients (67% men) and 94 caregivers (72% women) with an average age of 66.9 (SD = 7.4) and 56.3 years (SD = 14.1), respectively. There were significant differences for gender (*χ*^2^ = 30.6; *p* < 0.001) and age (t = −6.5; *p* < 0.001) between patients and caregivers. Approximately 46% of patients had a lower secondary diploma, while 48% of caregivers had an upper secondary diploma (*χ*^2^ = 16.3; *p* < 0.001). Most patients were retired or housewives (36%) or blue collar workers (engaged in manual labor) (35%), whereas 33% of caregivers were employees (workers engaged in non-manual labor) (*χ*^2^ = 17.2; *p* < 0.001). Most subjects in the total population were married, with no previous experience of psychological counseling and no psychiatric drug use (e.g., antidepressants, antipsychotics, anxiolytics, etc.). Individual psychotherapy had not been recommended to the majority of participants, whereas group psychotherapy had been recommended to about half. Among the patients, 61% of men had occupational exposure and 56% of women had environmental exposure to asbestos. There were also gender significant differences in average age (t = 3.5; *p* = 0.001), educational level (*χ*^2^ = 7.9; *p* = 0.048) and job (*χ*^2^ = 13.5; *p* = 0.004). The average age was 65.0 years (SD = 11.2) for men and 59.2 years for women (SD = 12.5). Most men (46%) had a lower secondary diploma, while 42% of women had an upper secondary diploma. Moreover, 38% of men were blue collar workers (38%), whereas 38% of the women were retired or housewives (Table 2).

The results of ANOVA analysis (Table 3) showed that MM patients and caregivers differ significantly only in DSQ Suppression and DTS scores. Specifically, MM patients reported a higher level of suppression (M = 5.1; SD = 2.2) compared to caregivers (F = 6.8; *p* = 0.010). However, the latter were more severely traumatized (F = 14.3; *p* < 0.001). 

Various statistically significant differences were observed between men and women in terms of behavior as well as coping strategies and defense mechanisms (Table 4). Women were more depressed (F = 13.5; *p* < 0.001) and traumatized (F = 25.2; *p* < 0.001) than men and used more coping strategies, particularly social support (F = 10.7; *p* < 0.001), avoidance (F = 3.9; *p* = 0.049), positive attitude (F = 4.6; *p* = 0.033), problem solving (F = 4.6; *p* = 0.032) and turning to religion (F = 6.0; *p* = 0.015). For defense mechanisms, women achieved higher scores than men for sublimation (F = 10.7; *p* < 0.00), anticipation (F = 4.9; *p* = 0.027), withdrawal (F = 26.6; *p* < 0.001), reaction formation (F = 7.0; *p* = 0.009), passive aggressive behavior (F = 9.8; *p* = 0.002), acting out (F = 5.7; *p* = 0.018), displacement (F = 8.1; *p* = 0.005) and somatization (F = 10.1; *p* = 0.002). On the other hand, men reported a higher level of dissociation than women (F = 4.8; *p* = 0.029).

There were 25 patients and 31 caregivers above the cutoff point for depression (BDI-II), and 12 patients and 30 caregivers above that for post-traumatic symptoms (DTS). The intergroup difference was significant only for post-traumatic symptoms (*p* = 0.042). Independent variables associated with BDI-II and DTS were examined by logistic regression analysis separately for patients and caregivers. 

In patients, a high risk of depression was associated with high use of DSQ Isolation (OR: 53.33; 95% CI: 3.22–882.30; *p* = 0.01) and DSQ Somatization (OR: 16.97; 95% CI: 1.04–275.90; *p* = 0.05). Conversely, high use of Coping–Turning to religion (OR: 0.003; 95% CI: 0.000–0.214; *p* = 0.01) and DSQ Humor (OR: 0.002; 95% CI: 0.000–0.286; *p* = 0.01) was found to have a protective effect against depression (accuracy of model: 88%, R^2^ = 0.684). In terms of effect estimates on post-traumatic symptoms, statistically significant risks were observed for DSQ Passive–aggressive behavior (OR: 24.88; 95% CI: 1.67–369.92; *p* = 0.02) and Coping–Avoidance strategies (OR: 165.87; 95% CI: 3.37–8173.81; *p* = 0.01), while DSQ Autistic fantasy (OR: 0.007; 95% CI: 0.000–0.414; *p* = 0.02) and Coping–Positive attitude (OR: 0.0001; 95% CI: 0.000–0.399; *p* = 0.03) had a potentially protective effect (accuracy of model: 92.6%; R^2^ = 0.660).

Among caregivers, women had an elevated risk of depression (OR: 6.39, 95% CI: 1.16–35.38; *p* = 0.03), especially those with a high use of Coping–Avoidance (OR: 8.93; 95% CI: 2.08–38.36; *p* = 0.003), DSQ Acting out (OR: 5.81; 95% CI: 1.34–25.08; *p* = 0.02), DSQ Devaluation (OR: 11.71; 95% CI: 2.27–60.45; *p* = 0.003), and DSQ Displacement (OR: 9.98; 95% CI: 2.17–46.00; *p* = 0.003). Meanwhile, a protective effect was observed in caregivers aged 61–69 years (OR: 0.10; 95% CI: 0.01–0.84; *p* = 0.03) and those with a high use DSQ Sublimation (OR: 0.08; 95% CI: 0.02–0.40; *p* = 0.002) (accuracy of model: 83%; R^2^ = 0.570). With regard to effect estimates on post-traumatic symptoms, a statistically significant risk was observed for DSQ Acting out (OR: 7.00; 95% CI: 1.10–44.47; *p* = 0.04) (accuracy of model: 81.9%; R^2^ = 0.565).

## 4. Discussion

The present study investigated the adaptive conscious/unconscious processes used to manage the emotional distress related to MM diagnosis in an Italian NPCS group of patients and familial caregivers. The sample adequately represented the incidence of the disease [1,4]. The results showed that patients and caregivers did not differ significantly with respect to the use of adaptational strategies, except for suppression. Emotional suppression is often used by cancer patients to not think about and verbalize the negative emotions related to cancer; nevertheless, there is evidence that this strategy seems to increase their levels of psychological distress [43,44,45,46]. This attempt to suppress unpleasant feelings and the idea of death has also been previously found in MM patients [13]. Thus, it could be possible that MM patients consciously decide to suppress thinking about the situation they are going through in order to regulate painful emotions they are not prepared to express.

Caregivers were more severely traumatized than MM patients, reporting a higher incidence of intrusive thoughts about death and physiologic hyperactivation. A possible cause is feelings of guilt, which is a key emotion in the experience of cancer caregivers that can compromise their psychosocial and somatic adjustment to cancer [47]. Caregivers in NPCSs are themselves exposed to asbestos and are thus at potential risk of developing and dying from MM. Hence, they may experience a form of survivor’s guilt for remaining healthy while their loved one(s) are expected to pass away [5,13,14]. Recent studies have consistently linked the emotion of guilt to post-traumatic symptomatology [48,49,50,51], suggesting a causal role for trauma-related guilt in this phenomenon [52,53].

Interestingly, gender appears to play a pivotal role in the understanding of both the emotional impact of the diagnosis of MM and activation of protective responses. Women showed higher levels of depressive and post-traumatic symptoms, as well as a more frequent use of many kinds of coping strategies and defense mechanisms, to face these negative experiences. Moreover, women caregivers were at greater risk than men of developing depressive symptomology, which is in accordance with the results of longitudinal studies demonstrating a higher risk of depressive disorders in women as compared to men [54,55].

Logistic regression analyses showed that adaptive processes were unconsciously activated in response to internal and external threats in both patients and caregivers at NPCSs. In fact, a few coping strategies partially explained the risk of developing depressive feelings, with avoidance strategies being a risk factor for caregivers and turning to religion serving as a protective factor in patients. Meanwhile, in patients, conscious strategies partially explained successive post-traumatic symptoms, with avoidance strategies and positive attitude as risk and protective factors, respectively.

Avoidance-oriented coping (i.e., avoidance, denial and withdrawal) is defined as the cognitive and behavioral attempts to distance oneself both from a problem and the related emotions [33,56]. These findings support the notion that avoidance strategies are the most frequent used to cope with MM-related emotional distress [9,10,14,15,57]. Nonetheless, the pervasive activation of avoidance strategies plays a pivotal role in the maintenance of post-traumatic symptoms [58,59,60] and increase their severity over time [61,62,63,64]. 

The protective effects of religious devotion may be attributable to its role as filter for aggression and anger [65]. Relying on religion allows patients to place their past, present, and future in a transcendent dimension [66,67], which may increase their hope and the feeling that they can manage their difficulties [68,69], provide comfort as they undergo chemotherapy and face its consequences as well as death, and explain their current situation [70,71]. From a psychoanalytic point of view, this can help people place their subjectivity at the center of their experiences, allowing them to tolerate the adversity that they face and prepare for death instead of seeking a target to blame. A positive attitude could be conceptualized as an adaptive coping strategy characterized by acceptation, the containment of painful emotions and a reinterpretation of a stressful event in a more positive way [32]. Our findings suggest that people who are able to positively change the value of thoughts related to the disease are less vulnerable to incur in post-traumatic conditions. It is possible that the efforts to assimilate—and not to avoid—intrusive negative thoughts and the traumatic emotions related to MM led to a more positive cognitive process of the cancer experience. Thus, maintaining optimism and positive expectations toward MM and treatments and believing that a stressful present can ameliorate in the future may restore a sense of self-efficacy and agency [10], promoting resilience and protecting from post-traumatic conditions [72].

Data suggest that defense mechanisms are important both for prevention and development of successive depressive or post-traumatic conditions. Psychoanalytic theories conceptualize defenses as a continuum, differing in degree of maturity, where primitive and/or immature mechanisms imply a severe alteration of reality testing, while more mature defenses allow for management of threatening feelings without distorting reality [73]. Our data support previous results showing that a pervasive use of immature defenses is a risk factor for the development of different forms of psychopathology [74,75,76,77]. For example, the use of immature defense mechanisms, such as somatization for patients, and acting out and devaluation for caregivers, increase the risk of incurring in depressive conditions, while the use of more mature defenses as humor for the patients and as sublimation for the caregivers, protect the Ego from depressive feelings. Sublimation could be conceptualized as a mature and adaptive mechanism allowing the subject to transform anxieties, aggressiveness, and inner conflicts, and thus restoring an inner balance [73]. It should be noted that similar results were also reported in previous research that took place in the same NPCS, identified as a resilient community able to transform unwanted feelings of rage, fear of death and desire of revenge into socially well-accepted claim actions for justice [13,76].

In line with this notion, we observed that immature defenses (i.e., passive-aggressive behavior in patients and acting out in caregivers) significantly increased the risk of developing post-traumatic conditions, although, surprisingly, autistic fantasy had a protective effect in patients. Autistic fantasy allows individuals to withdraw into a different state of consciousness so that they can temporarily distance themselves from external/internal stressful stimuli and their overwhelming impact [73]. In the short term, this defensive strategy can protect patients from the negative effects of a reality that is unmanageable on both psychological and behavioral levels; however, over time it can increase the risk of developing post-traumatic symptoms stemming from the inability to find meaning in the traumatic experience. Moreover, not only can avoidant defense mechanisms worsen post-traumatic conditions, the latter can promote the use of avoidance-coping strategies [78] on both emotional and behavioral levels. Indeed, people who are most severely traumatized by the psychological impact of MM frequently withdraw emotionally and socially [13], giving rise to a vicious cycle that can enhance their feelings of social isolation [18,79,80].

## 5. Conclusions

The findings of this study add to previous knowledge on psychological distress following an MM diagnosis by demonstrating that unconscious adaptive processes play a major role in the way patients and caregivers respond to overwhelming feelings related to the disease. Such evidence strongly suggests the need for psychological interventions addressed to both patients and caregivers, and that take into account not merely coping strategies, but also unconscious somatopsychic processes. In particular, from our clinical point of view, we believe that group interventions addressed to patients and caregivers together could constitute the better setting for the elaboration of unconscious processes connected to death anxieties aroused from MM, considering that the diagnosis has a traumatic impact on all the family members and on the relationship between them. Such interventions would be better time-limited, given the short life-expectancy for MM. Moreover, we suggest a somatopsychic focus for those interventions. Indeed, as suggested by our results, often both patients and caregivers rely on unconscious defense mechanisms to protect themselves from overwhelming emotions connected to the diagnosis, thus lacking a full and conscious comprehension of practical and affective consequences of the disease. Thus, a psychotherapeutic work with a somatopsychic focus can help restore the connection between the impact of physical symptoms and emotions, feelings, and fantasies related to the disease [77].

There were some limitations to our study. Firstly, we used only self-report measures that may have been influenced by social desirability biases. Secondly, since we did not collect any clinical data, we were unable to analyze our results based on disease stage or localization. Thirdly, since we considered MM patients and caregivers of the NPCS as specific subgroups, no generalization can be made regarding the whole MM population. Nevertheless, our study had a large sample size and used a variety of tests along with appropriate statistical models to identify relevant risk factors for, and protective factors against, psychological distress after a diagnosis of MM. Further research is needed to determine whether our findings are applicable to populations at other NPCSs.

## Figures and Tables

**Table 1:** Sociodemographic characteristics of patients (N = 108) and caregivers (N = 94).

Variables		Patients		Caregivers		*t*-Test; *p*-Value
		N	%	N	%	
Gender						
	Men	72	66.7	26	27.7	*χ*^2^ = 30.62; *p* < 0.001
	Women	36	33.3	68	72.3	
Age (mean ± SD)		66.9 ± 7.4		56.3 ± 14.1		*t*-test = −6.54; *p* < 0.001
Educational level						
	Primary school	18	16.7	9	9.6	
	Lower secondary school	50	46.3	28	29.8	
	Upper secondary school	23	21.3	45	47.9	*χ*^2^ = 16.29; *p* < 0.001
	Univerity degree	17	15.7	12	12.8	
Job						
	Blue collar workers	38	35.2	15	16.0	
	Employees	23	21.3	31	33.0	*χ*^2^ = 17.23; *p* < 0.001
	Entrepreneurs/Self-employed	8	7.4	20	21.3	
	Retired/Housewife	39	36.1	28	29.8	
Marital status						
	Married	83	76.9	76	80.9	*χ*^2^ = 0.48; *p* = 0.489
	Single *	25	23.1	18	19.1	
Psychotherapy						
	Individual	9	8.4	3	3.3	*χ*^2^ = 2.32; *p* = 0.13
	Group	46	43.0	42	46.2	*χ*^2^ = 0.20; *p* = 0.66
Previous psychological counseling						
	No	94	87.0	83	88.3	*χ*^2^ = 0.07; *p* = 0.79
	Yes	14	13.0	11	11.7	
Psychiatric drug use						
	No	86	79.6	79	84.0	*χ*^2^ = 0.65; *p* = 0.42
	Yes	22	20.4	15	16.0	

* Single + widower + separated.

**Table 2:** Sociodemographic characteristics of men (N = 98) and women (N = 104).

Variables	Men		Women		*t*-Test; *p*-Value
	M	SD	M	SD	
Age (mean ± SD)	65.0 11.2		59.2 12.5		*t*-test = 3.49; *p* = 0.001
Educational level					
Primary school	13	13.3	14	13.5	
Lower secondary school	45	45.9	33	31.7	*χ*^2^ = 7.91; *p* = 0.048
Upper secondary school	24	24.5	44	42.3	
Univerity degree	16	16.3	13	12.5	
Job					
Blue collar workers	37	37.8	16	15.4	
Employees	21	21.4	33	31.7	*χ*^2^ = 13.49; *p* = 0.004
Entrepreneurs/Self-employed	13	13.3	15	14.4	
Retired/Housewife	27	27.6	40	38.5	
Marital status					
Married	81	82.7	78	75.0	*χ*^2^ = 1.76; *p* = 0.184
Single **	17	17.3	26	25.0	
Psychotherapy					
Individual	7	7.2	5	4.9	*χ*^2^ = 0.47; *p* = 0.49
Group	42	43.3	46	45.5	*χ*^2^ = 0.10; *p* = 0.75
Previous psychological counseling					
No	89	90.8	88	84.6	*χ*^2^ = 1.79; *p* = 0.18
Yes	9	9.2	16	15.4	
Psychiatric drug use					
No	82	83.7	83	79.8	*χ*^2^ = 0.50; *p* = 0.48
Yes	16	16.3	21	20.2	
Exposure *					
Occupational	42	60.9	15	44.1	*χ*^2^ = 2.59; *p* = 0.11
Environmental	27	39.1	19	55.9	

* Only patients; ** single + widower + separated.

**Table 3:** ANOVA analysis results of patients (N = 108) and caregivers (N = 94).

Variables	Patients		Caregivers		F; *p*-Value
	Mean	SD	Mean	SD	
COPE * Social support	16.5	14.2	17.7	15.3	0.33; *p* = 0.564
Avoidance strategies	15.4	13.5	14.3	12.3	0.33; *p* = 0.564
Positive attitude	19.7	16.2	18.1	14.8	0.54; *p* = 0.462
Problem solving	17.7	15.0	18.6	15.6	0.16; *p* = 0.686
Turning to religion	13.7	11.0	14.5	11.5	0.24; *p* = 0.625
DSQ ** Sublimation	5.0	2.5	5.2	2.2	0.35; *p* = 0.553
Humor	5.2	2.6	5.4	2.2	0.36; *p* = 0.548
Anticipation	5.2	2.3	5.3	1.7	0.12; *p* = 0.733
Suppression	5.1	2.2	4.3	1.9	6.82; *p* = 0.010
Withdrawal	4.7	2.3	4.9	2.0	0.48; *p* = 0.489
Pseudo-altruism	4.7	4.4	4.5	1.8	0.08; *p* = 0.779
Idealization	4.4	2.3	4.5	2.3	0.04; *p* = 0.835
Reaction formation	4.6	2.1	4.9	2.0	0.68; *p* = 0.411
Projection	3.0	2.1	3.2	2.0	0.91; *p* = 0.343
Passive aggressive behavior	3.2	2.0	3.2	1.9	0.02; *p* = 0.891
Acting out	4.3	2.0	4.3	2.2	0.01; *p* = 0.948
Isolation	3.9	2.2	3.6	2.0	0.71; *p* = 0.402
Devaluation	4.5	2.1	4.3	2.0	0.38; *p* = 0.538
Autistic fantasy	3.5	2.6	3.1	2.3	1.36; *p* = 0.245
Denial	3.3	2.2	2.7	1.9	3.32; *p* = 0.070
Displacement	2.8	1.9	3.1	1.9	1.01; *p* = 0.317
Dissociation	3.7	2.4	3.3	2.0	1.95; *p* = 0.164
Splitting	4.1	2.0	3.9	2.2	0.40; *p* = 0.529
Rationalization	5.6	2.3	5.1	1.8	3.56; *p* = 0.061
Somatization	2.8	2.4	2.9	1.9	0.07; *p* = 0.799
BDI-II ***	9.7	8.9	11.4	0.9	1.99; *p* = 0.160
DTS **** Total Score	20.6	20.1	31.8	21.2	14.26; *p* < 0.001
Intrusion	6.0	7.7	10.1	8.1	13.15; *p* < 0.001
Avoidance/Numbing	6.5	7.6	7.7	6.0	1.39; *p* = 0.239
Hyperarousal	8.7	9.3	14.1	11.1	13.42; *p* < 0.001

* Coping Orientation to Problems Experienced – New Italian Version; ** Defense Style Questionnaire; *** Beck Depression Inventory, Second Edition; **** Davidson Trauma Scale.

**Table 4:** ANOVA analysis results of men (N = 98) and women (N = 104).

Variables	Men		Women		F; *p*-Value
	Mean	SD	Mean	SD	
COPE * Social support	13.7	12.8	20.3	15.7	10.70; *p* < 0.001
Avoidance strategies	13.0	12.2	16.6	13.5	3.92; *p* = 0.049
Positive attitude	16.6	14.9	21.2	15.9	4.59; *p* = 0.033
Problem solving	15.7	14.7	20.3	15.4	4.64; *p* = 0.032
Turning to religion	12.1	10.4	15.9	11.8	5.97; *p* = 0.015
DSQ **					
Sublimation	4.5	2.3	5.6	2.3	10.70; *p* < 0.001
Humor	5.4	2.5	5.3	2.4	0.77; *p* = 0.781
Anticipation	5.0	2.1	5.6	2.0	4.94; *p* = 0.027
Suppression	4.7	2.1	4.7	2.2	0.00; *p* = 0.987
Withdrawal	4.0	1.9	5.5	2.2	26.60; *p* < 0.001
Pseudo-altruism	4.3	4.5	4.9	1.9	1.34; *p* = 0.248
Idealization	4.1	2.3	4.7	2.3	3.26; *p* = 0.072
Reaction formation	4.3	2.0	5.1	2.0	6.99; *p* = 0.009
Projection	2.9	1.9	3.2	2.2	0.91; *p* = 0.342
Passive aggressive behavior	2.8	1.8	3.6	2.0	9.77; *p* = 0.002
Acting out	3.9	1.9	4.6	2.2	5.66; *p* = 0.018
Isolation	4.0	2.0	3.6	2.2	1.56; *p* = 0.214
Devaluation	4.5	2.1	4.3	2.0	0.71; *p* = 0.402
Autistic fantasy	3.5	2.4	3.3	2.6	0.30; *p* = 0.584
Denial	3.2	2.1	2.8	2.1	1.62; *p* = 0.204
Displacement	2.5	1.7	3.3	2.1	8.09; *p* = 0.005
Dissociation	3.9	2.3	3.2	2.1	4.83; *p* = 0.029
Splitting	3.9	1.9	4.1	2.3	0.58; *p* = 0.447
Rationalization	5.4	1.9	5.4	2.2	0.00; *p* = 0.996
Somatization	2.4	1.8	3.3	2.4	10.07; *p* = 0.002
BDI-II ***	8.2	8.5	12.7	8.7	13.49; *p* < 0.001
DTS **** Total Score	18.5	18.8	33.0	21.9	25.19; *p* < 0.001
Intrusion	4.7	5.9	11.0	8.8	35.37; *p* < 0.001
Avoidance/Numbing	5.5	6.4	8.5	7.1	10.08; *p* = 0.002
Hyperarousal	8.3	9.2	13.9	11.0	15.41; *p* < 0.001

* Coping Orientation to Problems Experienced – New Italian Version; ** Defense Style Questionnaire; *** Beck Depression Inventory, Second Edition; **** Davidson Trauma Scale.

## Data Availability

The dataset is available on request to the corresponding author.
